# Hybrid curation of gene–mutation relations combining automated extraction and crowdsourcing

**DOI:** 10.1093/database/bau094

**Published:** 2014-09-22

**Authors:** John D. Burger, Emily Doughty, Ritu Khare, Chih-Hsuan Wei, Rajashree Mishra, John Aberdeen, David Tresner-Kirsch, Ben Wellner, Maricel G. Kann, Zhiyong Lu, Lynette Hirschman

**Affiliations:** ^1^The MITRE Corporation, Bedford, MA 01730, USA, ^2^Biomedical Informatics Program, Stanford University, Stanford, CA 94305, USA, ^3^National Center for Biotechnology Information, National Library of Medicine, National Institutes of Health, Bethesda, MD 20894, USA and ^4^The University of Maryland, Baltimore County, Baltimore MD 21250, USA

## Abstract

Background: This article describes capture of biological information using a hybrid approach that combines natural language processing to extract biological entities and crowdsourcing with annotators recruited via Amazon Mechanical Turk to judge correctness of candidate biological relations. These techniques were applied to extract gene– mutation relations from biomedical abstracts with the goal of supporting production scale capture of gene–mutation–disease findings as an open source resource for personalized medicine. Results: The hybrid system could be configured to provide good performance for gene–mutation extraction (precision ∼82%; recall ∼70% against an expert-generated gold standard) at a cost of $0.76 per abstract. This demonstrates that crowd labor platforms such as Amazon Mechanical Turk can be used to recruit quality annotators, even in an application requiring subject matter expertise; aggregated Turker judgments for gene–mutation relations exceeded 90% accuracy. Over half of the precision errors were due to mismatches against the gold standard hidden from annotator view (e.g. incorrect EntrezGene identifier or incorrect mutation position extracted), or incomplete task instructions (e.g. the need to exclude nonhuman mutations). Conclusions: The hybrid curation model provides a readily scalable cost-effective approach to curation, particularly if coupled with expert human review to filter precision errors. We plan to generalize the framework and make it available as open source software.

**Database URL:** http://www.mitre.org/publications/technical-papers/hybrid-curation-of-gene-mutation-relations-combining-automated

## Background

The fields of translational medicine and personalized medicine rely on coupling information about an individual’s genotype (genetic make-up and genetic variations) with their phenotype (physical characteristics and state of health/disease). Recent advances in low-cost rapid genomic sequencing methods now enable population studies linking genotype and phenotype. Several large-scale research activities are underway, including the eMERGE Network (electronic Medical Records and Genomics, http://emerge.mc.vanderbilt.edu/emerge-network), and the Personal Genome Project (1). This approach is making it possible to identify a patient’s genetic variations that are associated with a particular disease state or drug response. Similarly, new findings in pharmacogenomics are enabling more precise dosing, for example, for warfarin (2). However, to make it possible to translate these associations into improved patient care, it is critical to have ‘an authenticated, well-annotated, curated, and freely accessible knowledge base of genomic associations, risks, and warnings in machine-readable form’ (3). The focus of this research was to explore ways to create such a comprehensive, up-to-date, freely accessible knowledge base of genetic variations and associated phenotypes.

### The curation problem

There is an increasing volume of gene–mutation–disease information being published in the biomedical literature. We estimate that there are >10 000 new articles published each year that describe findings on the relation between genetic mutations and phenotype or diseases. There are multiple repositories for this information, including OMIM (4), dbSNP (5), HGVbase (6), HGMD (7), PharmGKB (8) and locus-specific databases (9). However, until recently, there have been few publicly available curated resources that capture such findings. The recent creation of National Center for Biotechnology Information’s (NCBI’s) ClinVar database (10), launched in 2013, now provides a single public repository for such information. As of December 2013, ClinVar had >80 submitters and >60 000 variations submitted.

There are currently two main approaches to capture computable genomic information: author-deposited data and expert curation. Author-deposited data (such as found in dbSNP or ClinVar) has the advantage in terms of cost (because the cost is borne by the researcher) and expertise (clearly the discovering researcher is in the best position to ensure accurate recording of the information). However, there are corresponding downsides: authors may not want to take the time to record their findings in detail, and they may not have the cross-disciplinary bioinformatics expertise to link findings to the underlying biological reference databases. For the capture of gene–mutation–disease relations, for example, the author must provide EntrezGene identifiers, mutation position information based on a reference genome, and the appropriate Medical Subject Headings (MeSH) or Unified Medical Language System (UMLS) term for the disease or phenotype. In an experiment to compare author-contributed findings with expert curation in the MINT (Molecular INTeraction) database, author recall on curating protein–protein interaction was reported at 57% compared with curator recall of 87%, measured against a curated gold standard (11).

Expert curation is the norm for reference biological databases, such as for the Mouse Genome Informatics (MGI) database (12) or TAIR (The Arabidopsis Information Resource) (13); expert curation of findings from the literature provides high-quality annotations, but throughput (and therefore coverage) is limited by cost (14). There are alternatives to these approaches, for example, automated capture of information through bioinformatics methods. MGI applies automated annotation of gene– protein function as a first pass, for cases where expert curation has not yet been done (these automated annotations are labeled with a specific evidence category, to distinguish them from the expert curated annotations). Another approach that is gaining acceptance is crowdsourcing or community-generated annotations (14, 15). These techniques are being leveraged to collect data on drug interactions, drug adverse events and other complex relations reported in the literature. Other researchers (16) have experimented with crowdsourcing to create gold standard datasets for evaluation of biomedical curation tasks.

### Scaling the curation process

The goal of this research has been to create a scalable cost-effective curation process to capture complex information from the literature and make it available to the research community in a timely fashion, at an acceptable level of accuracy. We can posit throughput and cost goals; however, it is more difficult to determine accuracy requirements, except by comparison with existing curation efforts.

When depositing curated information into a database, high precision is critical to avoid depositing incorrect information. An early study on inter-curator agreement for Gene Ontology (GO) curation (17) reported that expert GO annotators achieved 91–100% precision, and at least 72% recall, micro-averaged over results from three curators. A more recent study of curation by the Comparative Toxicogenomic Database (CTD) team (18) reported similar results on a complex curation task for disease–gene– chemical relations from the literature: 91% of the interactions extracted by CTD biocurators were judged by the lead curator to be correct, with an average recall of 71%. These results provide a target performance of at least 90% precision at a recall of at least 70%. An alternate strategy would have been to optimize a semi-automated workflow for recall, followed by expert review to remove false positives. This approach is revisited in the Discussion section.

To achieve the goal of scalable, timely, cost-effective, accurate curation, our targets for the hybrid curation workflow were as follows:

- To provide accuracy comparable with expert curated databases, namely, at least 90% precision with recall of 70% (17, 18);
- To keep up with the volume of literature (∼10 000 abstracts per year or 200 abstracts per week);
- To be affordable—we picked a cost of $1 per abstract as a target for simple gene–mutation relations; full gene– mutation–disease curation could be an order of magnitude more complex, but even a cost of $10 per abstract for full curation would be an affordable price to pay to make the findings computable and reusable, compared with the cost of publication for an open access article, often ≥$1000.

## Approach—combining automated extraction and curation

Our approach has been to combine automated extraction of the relevant biological entities, namely genes and mutations, with crowdsourcing to elicit human judgments on the correctness of associations between entities. The general strategy was to have high recall on the automatic entity extraction, followed by human judgment for all possible pairwise gene–mutation combinations, with the final goal of high precision at the relation level, to support deposit of the extracted relations into an open source repository after expert review.

**Figure 1.**
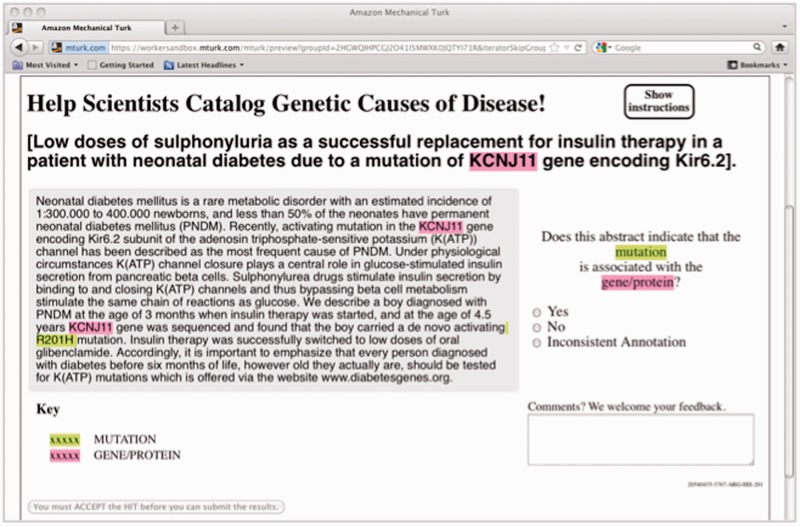
HIT design for the gene–mutation task.

For this experiment, the genes were extracted using the augmented GenNorm system (19) to identify each mention of a gene and associate it with the appropriate EntrezGene identifier. The mutations were extracted using the Extractor of Mutations system (Extractor of Mutations, EMU) (20). Each candidate gene–mutation pair in an abstract was then presented as highlighted mentions in a MEDLINE abstract and passed on to a crowd labor platform, where workers were asked to judge whether the highlighted gene was associated with the highlighted mutation (Figure 1).

In this work, we made use of a crowdsourcing platform called Amazon Mechanical Turk (MTurk). MTurk is a web-based labor market for what Amazon calls *Human Intelligence Tasks* (HITs). A typical HIT is a minimal problem instance that cannot be easily automated, such as categorizing a snippet of text or an image. Most HITs take a worker (or *Turker*) only a short time (seconds) to complete, and workers are paid on a piecework basis. In 2011, Amazon reported that 500 000 Turkers from 190 countries were registered on the service (21).

## Methods

### Overall experimental design

We applied this framework to extract gene–mutation relations from titles and abstracts of articles from PubMed and evaluated the results by comparison with an expert-curated gold standard of gene–mutation–disease relations. Building on results from an earlier experiment (22), we refined the framework and enriched the gold standard for easier evaluation.

**Figure 2.**
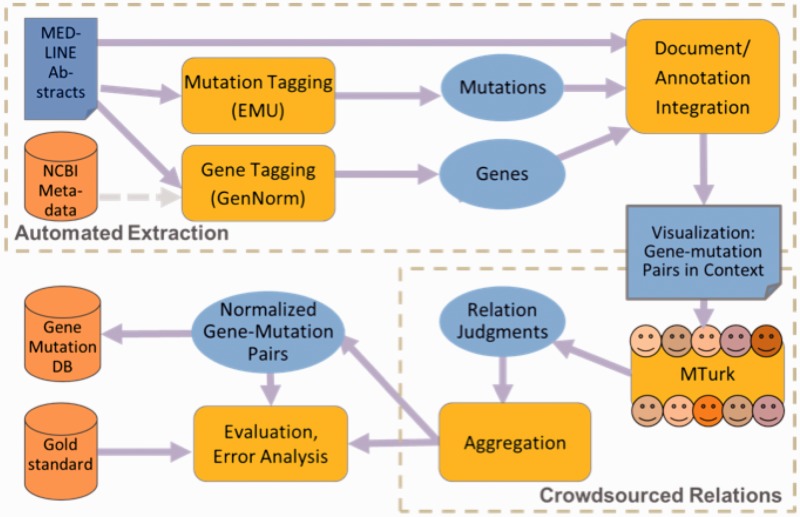
Schematic framework for hybrid curation.

A schematic of the framework is shown in Figure 2. The software combines multiple modules to support the following functions: automated information extraction, using the NCBI’s GenNorm and the University of Maryland Baltimore County’s (UMBC’s) EMU; linkage of multiple mentions of each unique gene and each mutation; display of each candidate gene and mutation pair, highlighted in context in the abstract; crowdsourced relation judgment (via Amazon MTurk); and evaluation against a gold standard.

In the automated extraction phase, for the titles and abstracts of documents (in MEDLINE format), we identified and normalized as stand-off annotations:

1. mentions of genes, associated with EntrezGene ids, using NCBI’s GenNorm tool (this differs from the initial experiment, which relied on NCBI-provided gene annotations in gene2pubmed), and
2. mentions of mutations, identified and encoded as wild type, mutation type and position by a modified and updated version of UMBC’s EMU tool (as in our initial experiment).

We then integrated the output of the entity extraction tools and the source signal (the text of the MEDLINE abstract), grouped the text mentions by concept identifier into sets of unique mutations and genes and then took the cross product of mutation and gene concepts within each abstract, producing all potential gene–mutation pairs as candidate relations.

In the crowdsourcing phase, we used human judgment via the MTurk platform to filter the candidate relations down to those that are supported by the text of the abstract. We presented each concept pair as an MTurk HIT, highlighting the gene and mutation mentions in the context of the abstract (see Figure 1), and asked workers who have passed an initial qualifying exam to judge whether the candidate relation is accurate. For each candidate relation, we obtained judgments from five Turkers.

Finally, we evaluated the accuracy of the entity extraction and the relation judgments, as well as the combined accuracy across the full pipeline. The Turkers’ relation judgments were evaluated both individually and in aggregate across each candidate according to a variety of aggregation strategies. Each of these components is described in further detail below.

### Data sources and gold standard

We used the curated gene–mutation–disease corpus described in Doughty *et al*. (20) covering breast cancer and prostate cancer, augmented by additional curation to include abstracts covering autism spectrum disorder. Abstracts were downloaded from the PubMed search engine using the MeSH terms ‘Mutation’ (for breast and prostate cancer) or ‘Mutation AND Polymorphism/genetic’ (autism spectrum disorder). The downloaded abstracts were then further filtered to include only those in which the EMU mutation extraction tool identified at least one mutation mention, resulting in a corpus of 810 abstracts.

Curators with domain expertise then annotated the 810 abstract corpus to create a gold standard. The curators identified human gene mutations mentioned in each abstract and whether those mutations were mentioned in relation to one of the three diseases under study; the gold standard included annotation for single nucleotide polymorphisms (SNPs) associated with disease in humans. All gene–mutation–disease relations were marked as coding or noncoding; all mutations that did not involve human genes were marked with a special code; each mutation position was specified in terms of nucleotide or protein position within a reference gene. Although the initial curated data set created at UMBC covered gene–mutation–disease relations, the focus of the experiments described here was on the gene–mutation relations for SNPs within coding regions of human genes.

At the outset, we split the corpus into three segments. For the initial experiment, we used a subset of 250 abstracts that had already been curated for genes in PubMed because at that time we did not have access to an automatic gene annotation tool. We then randomly split the remaining abstracts into two sets of 275 abstracts, one of which was used in the experiment described here. The final set of 275 documents has been held in reserve for a future experiment. The remaining 10 abstracts were used for detailed debugging during development. Distributions for genes and mutations are shown in Table 1. The differences between the two data sets in Table 1 were most likely owing to the way in which we chose the first set, which resulted in a bias toward more recent articles, leaving older articles with fewer mutations per article, in the second set and third unused set.

**Table 1.** Corpus comparison for Experiment 1 vs. Experiment 2

Experiment	#1	#2
Number of Abstracts	250	275
Number of HITS	1097	1078
Number of genes (gold standard)	279	246
Number of mutations (gold Standard)	586	452
Number of gene–mutation pairs (gold standard)	586	444
Gene–mutation pairs per abstract	2.3	1.6

### Extraction of base entities (genes, mutations)

#### Mutations—extracted by EMU

To identify and standardize mutation mentions in the target documents, we used the EMU (20). EMU has a two-step filter process to identify SNP mentions: first, the text was searched for matches to a list of regular expressions, and each match in the text was parsed leveraging the internal component structure of the regular expression that identified it; second, the matches were filtered using a different group of regular expressions as a stop-list. Substrings identified as mutations had surface representations identifying them variously as nucleotide substitutions (‘c.758C>T’), amino acid substitutions (‘Arg 117 His’) or database identifiers (‘rs35490896’). EMU standardized compositionally described mutation mentions (e.g. ‘c.758C>T’) by parsing to extract the values of a wild type, a mutated type and an integer position for the mutation. However, no standardization was performed for mentions that used semantic identifiers of SNPs (e.g. ‘rs35490896’). For cases where EMU detected pairs of mutant and wild-type nucleic acids or amino acids, it searched the local environment for positional information; for example, the phrase ‘T to C at position 59’ produced the triple (T,C,59) of type ‘nucleotide’.

We made several adaptations in our use of EMU in the current experiment:

- The version of EMU used in these experiments (version 1.19) was capable of identifying various types of mutations, but this work focused on SNPs in coding regions; the processing therefore filtered out noncoding mutations from EMU output by excluding mutations that had negative positional information or were explicitly stated to be in introns or untranslated regions.
- EMU in its released versions identified only one mention for each mutation concept within a document. The downstream representation of candidate relations for judgment by Turkers relied on identification of all mentions, to highlight them in context. Accordingly, we modified EMU to report multiple mentions for each mutation concept, with stand-off annotation indicating the text position of each mention.
- EMU was packaged with a postprocess filter that compares mutation–gene candidate pairs against a reference genome to determine whether the wild type and position are credible. Our experiments were designed with the intent of high recall in the entity extraction phase, so we did not apply this postprocess.

#### Genes—extracted by GenNorm

In the initial experiment (22), we relied on gene metadata present in the MEDLINE record (indexed by NCBI), and found gene mentions (GMs) by projecting the indexed genes into text using a database of gene names and simple heuristic string matching. However, for the current experiment, we used a modified version of the GenNorm gene tagger that operated directly on the text.

GenNorm (19) is an algorithm for assigning NCBI Gene identifiers to gene and proteins mentioned in biomedical text; it was a high-performing system as evaluated in the BioCreative III Gene Normalization task in 2010 (23, 24). GenNorm consists of three modules for (i) finding GMs, (ii) identifying and assigning organism information to GMs and (iii) normalizing mentions to NCBI Gene identifiers.

For this study, the gene name lexicon used in the normalization module was downloaded from the NCBI Gene FTP site on 19 November 2012. Because this study only focused on mutations in human genes, we modified the species recognition module of GenNorm such that only human genes were returned. Two additional steps were implemented to optimize results for this study:

First, a second gene tagger was included. The original GenNorm uses AIIA-GMT (25) for recognizing GMs from a textual passage. To maximize recall, we added another system, GeneTuKit (26), to the GM module of GenNorm. Based on the benchmarking results on the BioCreative II Gene Normalization task (27) test data, we observed ∼5% increase in recall.

Second, an abbreviation resolution module was included. Based on our error analysis of GM recognition results, we found that certain mentions identified by the GM module were not human genes or proteins. For example, from the phrase ‘chloramphenicol acetyltransferase (CAT)’, our system mistakenly recognized the abbreviation ‘CAT’ as a GM because it matched with the official symbol of the human gene ‘CAT catalase’ (NCBI Gene ID: 847), even though the associated full gene name ‘cloramphenicol acetyltransferase’ does not refer to a human gene. To filter such false positives, we applied a biomedical abbreviation resolution tool, AB3P (28), which identifies pairs of long and short forms from sentences. For example, given the statement ‘Antagonists selective for estrogen receptor alpha (ESR1)’, the tool would recognize the pair 〈estrogen receptor alpha, ESR1〉. Using the AB3P output, we were able to validate the results of our normalization module as follows: a short form (and its identifier) was kept in the GenNorm output only if the whole or part of its long form corresponded to a human gene.

### Building gene–mutation candidates

The automatic extraction processes described above typically identified mentions of multiple genes and mutations in each abstract, but we wished to show the human annotators only one gene and one mutation at a time (albeit with potentially multiple mentions of each). We automatically grouped multiple mentions of the same gene in a single abstract using the gene ID assigned by GenNorm. Similarly, we grouped multiple mentions of the same mutation using the standardized form produced by EMU, namely, a triple of 〈position, wild type, mutant type〉, where the wild and mutant types were either both nucleotides or both amino acids. One abstract contained a reference to a mutation via a dbSNP reference id (e.g. ‘rs1234’). We ignored this reference in calculating our results, as dbSNP references are already associated with a specific gene in the dbSNP database. Finally, the cross product of all distinct genes and mutations found in an abstract was formed, resulting in 1078 distinct gene–mutation items for the annotators.

### Turking

Just as in our earlier experiment (22), each item was presented to the Turkers with all the mentions of the gene and mutation highlighted, to enable quicker annotation. Figure 1 displays a screen shot of one such item as the Turkers saw it. The Turker had to decide whether the highlighted gene and the highlighted mutation were described in context as related to one another (‘Yes’ or ‘No’), with an additional option for indicating that the annotations are problematic in some way (‘Inconsistent annotation’). In practice, this third option was rarely chosen (only 1.4% of the responses).

Because Turkers are not individually vetted for any particular task, requesters use a variety of mechanisms to discourage poor performers from participating. The simplest approach is to require a specific minimum approval rating on their previous work, whether for the same or other requestors; in this study we required Turkers with at least 95% rating on previous tasks. We also made use of a task-specific pretest or *qualifier*, consisting of five sample HITs with known answers, that Turkers had to pass (with four of five, or 80%, correct) to work on our items. This was similar in form to the item shown in Figure 1, and was composed of five distinct candidate gene–mutation relations (the test items for the Qualifier are shown in Supplementary Appendix D). These were gleaned from our initial experiment and were the five most predictive items of Turker performance in that experiment measured by linear correlation.

We also used a more dynamic measure of Turker efficacy by injecting *control items* into the workflow. Approximately 20% of the items that each Turker performed were drawn from a separate set of 99 items that were also predictive of Turker performance in our initial experiment. These were the successive items in the correlation ranking described above, chosen for a near-balance of positive and negative.

Finally, we used a common technique for quality control, namely, redundancy. We requested that five distinct Turkers complete each item, enabling voting and other more sophisticated response aggregation strategies, as we describe below.

### Evaluation methodology

The combined machine–human workflow discussed in this article produced various kinds of artifacts at a number of distinct levels, and we were interested in evaluating these separately and in different ways. The most obvious dimension was that of entities vs. relations, where the entities of interest included genes and mutations, and the relation we were after was the one that held between genes and mutations. Another important dimension was that of surface text vs. underlying concepts. In our case, the concepts were represented by NCBI identifiers for genes, and the wild– mutant position triples for mutations. The gene–mutation relation was then simply represented by tuples of gene and mutation identifiers. Table 2 shows these two dimensions graphically.

**Table 2.** Dimensions of possible evaluation

Element of analysis	Displayed surface text (Turker view)	Concepts (Database view)
**Entities**	1. Gene spans; mutation spans	2. EntrezGene IDs; mutation triple
**Relations**	3. Judgments on entity spans in context	4. Tuple of 〈gene ID, mutation triple〉

The distinction between *Displayed Surface Text* and *Concepts* is worth making to better understand the mistakes made in different parts of our processing pipeline. In particular, the Turkers only saw the representation of genes and mutations as highlighted in the text, and so should have been evaluated on what they were shown (Quadrant 3 in Table 2). Ultimately, we imagined this workflow depositing gene–mutation relations into a database in terms of the underlying concepts (Quadrant 4 in Table 2), and it was equally important to evaluate the efficacy of this process. However, the automated system may have correctly recognized a gene and a mutation mention in an abstract, but could still assign the wrong concept ID to one or both. In this case, the Turkers may have correctly identified an instance of the relation, even though it would ultimately be a mistake to deposit this relation into a database. Thus, we wished to evaluate these two quadrants separately.

As shown in Table 2, Quadrant 1 corresponded to the gene and mutation spans found by the automatic components at the beginning of our pipeline. This quadrant could be evaluated using metrics designed for mention-level tagging, as done for the gene name task in the first two BioCreative evaluations (29, 30), but we did not do so here because it would have required the preparation of a special set of annotations that included all mentions of genes and all mentions of mutations, regardless of their association to a mutation or disease.

Quadrant 2 represented the gene and mutation concepts, and we were in fact interested in evaluating these because they formed the basis for our ultimate interest, the relations between them. We wished to understand, for instance, the degree to which errors in the entities affect errors in the relation level. This evaluation corresponded to the determination of whether a particular gene or mutation concept was mentioned in an abstract, and we used the UMBC gold standard discussed above for this. However, as the gold standard was curated for gene mutations with possible association to disease, if there were genes mentioned in the abstract that were not associated with a mutation or disease, they would not appear in the gold standard. For these reasons, we focused on recall (and not on precision) for Quadrant 2 results (see Table 3), as failure to capture a gene or mutation meant that the Turkers would never see the candidate relation to pass judgment on it.

**Table 3.** Precision and recall scores for gene and mutation identification

Element of analysis	Gold standard	Candidates	Correct	Precision	Recall
Genes	246	582	222	0.381	0.902
Mutations	452	497	395	0.795	0.874
Gene–mutation pairs	444	1078	374	0.347	0.842

Quadrant 3 corresponded to the task performed by the Turkers, namely, given sets of gene and mutation spans in an abstract, to judge whether the context indicated that the relation in question held. We could not use the UMBC gold standard for this because it was expressed in terms of gene and mutation concept IDs. We undertook several experiments using staff from MITRE, UMBC and NCBI to annotate subsets of the HITs shown to the Turkers, independent of the gold standard, to provide a baseline against which to compare the Turkers; this enabled us to estimate Turker accuracy on this task; see Supplementary Appendix A for the details of these experiments.

Finally, Quadrant 4 was the relation concept representation. This was the end product of the workflow, from which the system would ideally deposit validated relations into a database. We evaluated performance using the UMBC gold standard directly. If a 〈gene ID, mutation triple〉 was present in the gold standard for a particular abstract, we declared it to be correct, and otherwise it was incorrect. This quadrant provided the main results of the article, which are presented in the next section.

## Results

### Base entity extraction results

We measured how well the preprocessing captured the relevant mutations and genes by comparing them against the sets of mutations and genes that appeared in the gold standard curated gene–mutation associations. Table 3 shows the actual number of entities in the 275 abstracts in our dataset, the number of candidates identified by GenNorm and EMU and the number that were correct. From this we calculated precision and recall for the two types of entities. (For the reasons discussed in the *Evaluation Methodology* section, these numbers should be seen as close approximations to actual precision and recall, as there may have been a few mutations or genes not included in the gold standard used for scoring.) Note that recall was higher than precision in both cases—in our pipeline it was preferable to over-generate candidates, as we relied on the Turkers to improve precision downstream.

### End-to-end results

The experiment was posted to MTurk on 12 December 2012, comprising 1354 items, of which 20% were controls drawn (with replacement) from the 99 items as described above. Because we asked for five Turkers to respond to each item, we received 6770 judgments overall, from 24 distinct Turkers. The most prolific Turker produced judgments for all 1354 items, while the least prolific Turker produced only one. Turkers were paid 7¢ per judgment, for a total cost of $521.29, including Amazon’s overhead charges. The last judgment was received almost exactly 11 days after posting. At >600 judgments per day, this was a satisfactory throughput rate, although substantially slower than the 36 h recorded for our first experiment (22), perhaps because of the timing of the December holidays.

#### Concept relation accuracy

Table 4 shows the concept relation accuracy for the Turker judgments (corresponding to Quadrant 4 from Table 2) for both the initial experiment (Expt 1) and the current experiment (Expt 2). The concept accuracies were evaluated using the UMBC Gold Standard. The per-Turker accuracies were lower than the per-item accuracies owing to a number of poor-performing Turkers who submitted only a handful of judgments (seven Turkers submitted l<10 responses each). As we restrict our attention to those who performed larger amounts of work, we can see increases in performance. We also see that the results for Experiment 2 were lower than those for Experiment 1, despite use of a qualifying exam in Experiment 2 and careful selection of control items. These findings led to several detailed error analyses presented in the Discussion section.

**Table 4.** Concept relation accuracy for the initial experiment vs. the current study

	Individual Turker results	
	Experiment 1 %	Experiment 2 %
Baseline system (all ‘NO’)	58.7	65.6
Average response	75.5	73.7
Average 10+ Turker	70.7	68.1
Average 100+ Turker	76.0	75.8
Best Turker	90.5	88.8
Naïve Bayes aggregate	84.5	85.3

#### Aggregate results

One way to compensate for the poor-performing Turkers is to aggregate judgments on the same item—this was the main reason for requesting multiple judgments. There are a variety of possible mechanisms for such combination, and we explored several in previous work, with the most promising being the Naïve Bayes algorithm (31). Control item performance correlated roughly with test item performance (see Supplementary Appendix B), so we used the former as the basis for each Turker’s Bayes factor. In brief, each Turker corresponded to a separate Naïve Bayes feature, with the Bayes factors calculated with respect to their accuracy on the control items. The first line of Table 5 (85.3%) shows the accuracy resulting from combining all five responses for each test item using Naïve Bayes. By comparing this with the ‘average response’ line of Table 4 (73.7%), we can see that aggregating provided a substantial improvement, almost 12 points of accuracy. Essentially, Naïve Bayes has discovered the good performers and weighted their contributions substantially more highly than the poor performers.

**Table 5.** Concept accuracy using Naïve Bayes aggregation, varying number of Turkers

Number of Turkers	5	4	3	2	1	Dynamic
Concept accuracy	85.3%	84.2%	82.7%	81.6%	74.3%	86.0%
Cost	$1.89	$1.51	$1.13	$0.75	$0.38	$0.97

We incurred a substantial premium for this improvement in performance, however, paying five times as much as the single Turker case for the opportunity to aggregate multiple responses. The remainder of Table 5 suggests the aggregate performance we would get from asking for fewer and fewer judgments per item. We simulated this by simply removing the last Turker(s) to respond to each item. We can see that even with only double redundancy (*n* = 2) we gained a great deal using response aggregation (81.6%).

#### Results for ‘active management’ simulations

In addition to statically asking for a constant number of Turkers per item, as shown in Table 5, we also simulated managing the Turkers more dynamically. Based on control item performance, we would ‘fire’ Turkers when they appeared to show little promise. In particular, Turkers who were performing below chance (50% for the control items) were good candidates for early dismissal. We simulated this after the fact in the results below, although in separate work we have experimented with automatically blocking poor Turkers dynamically while work is being performed.

We can see that the dynamic approach was almost as inexpensive as two Turkers per item, and accuracy was actually higher than the five Turker case (86%). This is because the Naïve Bayes aggregation gave some nonzero weight even to poor performers, while cutting those Turkers off early allowed us to ignore their contributions entirely.

### Turker surface accuracy

As explained in the preceding section, we wished to assess the various parts of our pipeline separately. In particular, we wanted to distinguish between errors made at the surface judgment level by the Turkers (Quadrant 3) and errors at the final concept level (Quadrant 4). We used the UMBC gold standard to assess the latter, but this was in terms of concept IDs, which were invisible to the Turkers. To assess the Turker judgments directly, we would ideally have used a gold standard that ignored whether the concept IDs were correct. We made several attempts to construct this, with varying degrees of success. Our best attempt at a surface-level gold standard was accomplished by performing the Turker task ourselves on a subset of the items, notably those in which the aggregate Turker judgment disagreed with the UMBC gold standard at the concept level. This was done by three authors (J.A., L.H., R.K.); where all three authors agreed, we used this to rescore the surface relation results, but otherwise left the results as they were. Full details are provided in Supplementary Appendix A. (Note that this arguably provided an optimistic estimate, as it could only remove Turker-Gold standard disagreements, not discover new ones).

Table 6 shows this estimate at surface accuracy, together with the concept accuracy from above for comparison. We also show several other figures of merit. We can see that from the perspective of what they were asked to do, the Turker aggregate performed well.

**Table 6.** Surface and concept aggregate performance

	Surface level—Quadrant 3	Concept level—Quadrant 4
Accuracy	90.6	85.3
Precision	83.6	71.9
HIT recall	95.1	94.3
End-to-end recall	91.7	78.8

#### Surface and concept relation accuracy

Table 6 also shows the end-to-end concept relation accuracy for the Turker judgments (corresponding to Quadrant 4 from Table 2). The concept accuracies were evaluated using the UMBC Gold Standard. HIT recall computed recall against the HITS corresponding to gold standard entries; end-to-end recall computed recall against the entire gold standard (including relations not extracted in the automated processing).

### Interannotator agreement

We explored interannotator agreement among the most prolific Turkers. Because the MTurk platform allowed Turkers to do as few or as many HITs as they liked, calculating interannotator agreement over all Turkers who participated was not feasible. Thus, we calculated pairwise agreement and pairwise Cohen’s Kappa (32) for the three Turkers who did >1000 HITs. Pairwise raw agreement (% of responses in common) and Cohen’s Kappa for each pair of Turkers appears in Table 7.

**Table 7.** Pairwise agreement and Kappa for each pair of most prolific Turkers

	A–B	A–C	B–C
**% Agreement**	0.630	0.680	0.731
**Cohen’s Kappa**	0.263	0.378	0.477

## Discussion

### Comparing Turker performance in two experiments

Following the completion of this second experiment, we compared the concept relation accuracy for the Turker judgments for both the initial experiment and the current experiment, as shown in Table 4. To our surprise, despite the more careful vetting of Turkers and more careful selection of control items, the concept accuracy of this second experiment was comparable with the first experiment (84.5% for experiment 1 vs. 85.3% for experiment 2). These findings led us to do several detailed error analyses.

### Post hoc error analyses: false positives

Table 8 shows the gold standard vs. Turker judgments under two sets of conditions: first, for the full set of HITs, and in the adjacent column in parentheses, for a subset of HITS (explained below). For the full set of HITS, there were 137 false positives of 487 (HITs where the aggregate Turker judgment was YES, but the gold standard said NO), resulting in a precision of 71.9%, far below the 90% precision that we deemed necessary for a production quality system.

**Table 8.** Turker aggregate judgment vs. gold standard

Naïve Bayes aggregate score	GoldStd YES		GoldStd NO		Total		Percent		Concept level Eval
	All HITs	*Local Pos*	All HITs	*Local Pos*	All HITs	*Local Pos*	All HITs	*Local Pos*	
Turker YES	350	*(317)*	137	*(68)*	487	*(385)*	71.9	*(82.3)*	Precision
Turker NO	21	*(20)*	570	*(447)*	591	*(467)*	94.3	*(94.1)*	HIT recall
Turker Total	371	*(337)*	707	*(515)*	1078	*(852)*	85.3	*(89.7)*	Accuracy
TOTAL GOLD					444	*(444)*	78.8	*(71.4)*	E2E recall

On further analysis, we noticed that many of the false positives resulted from mismatches between the HIT shown to the Turker (the Quadrant 3 Turker view, Table 2) and the concept level view of the gold standard (the Quadrant 4, Table 2) that was not visible to the Turkers.

Specifically there were a number of discrepancies owing to an incorrect mutation position value. This happened when EMU had to look for positional information that did not occur as part of a standard type of mutation expression, as in ‘… T to C at position 59 resulting in substitution of Pro for Ser’. Here EMU correctly identified Pro and Ser as the mutant and wild-type amino acids, but picked up the closest number as the position (‘59’) that was incorrect—it was the position at the nucleotide level, not at the amino acid level. The position information that EMU found in this way was *not* highlighted when displaying the HIT; thus, the Turker may have seen a gene–mutation relation that was correct at the surface level, even though the underlying representation at the concept level did not match the gold standard because of erroneous position information. The EMU output included a set of 120 mutations with ‘non-local’ position information. Of these, 82 of 120 (68%) had incorrect positional information.

Based on this analysis, we simulated *disallowing* the processing of nonlocal mutations within EMU by removing the 226 HITS associated with the nonlocal mutations. We then rescored; the revised results, shown in the columns labeled ‘*Loc Pos*’ showed a HIT accuracy of 89.7%, with a precision of 82.3% (false positive rate of 17.7%) and HIT recall of 94.1% (false negative rate of 5.9%). However, end-to-end recall decreased substantially, from 78.8 to 71.4% because of removal of a number of valid HITs.

#### Categorization of the remaining false positives

We next undertook a detailed analysis of all the HITs where the aggregate Turker judgment differed from the gold standard, after removing the problematic mutations described above. We analyzed the remaining 68 false positives to identify other types of mismatches and information hidden from the Turkers. We identified a number of types of mismatch, including erroneous EntrezGene identifier (21 occurrences), inclusion of nonhuman or noncoding mutations (23 and 11 occurrences, respectively), missing gold standard information (3 occurrences) and erroneous Turker judgments (10 occurrences). The details of this analysis are provided in Supplementary Appendix C.

Overall, almost two-thirds of the false positives were the result of errors hidden from the Turkers (wrong gene concept identifier) or failure to give full instructions to Turkers (to exclude nonhuman mutations). We concluded from this analysis that the aggregate Turker judgments were surprisingly good, especially if judged in terms of surface relations. This was corroborated by our attempt to measure Surface Relation Accuracy (Quadrant 3) as described in Table 6. Based on these results, we estimated that aggregate Turker judgments were >90% accurate.

#### Categorization of false negatives

Table 8 shows two recall figures. The first (higher) of these was the ‘HIT Recall’; this was the recall in terms of only those candidate relations that the Turkers were presented with. The second (‘End-to-end recall’) measured the recall of the entire hybrid process, including both omissions due to faulty extraction of genes or mutations, as well as incorrect Turker judgments. There were relatively few false-negative Turker judgments (on the order of 5–6%). The larger problem occurred during candidate entity extraction, due to failure to extract gene identifiers or full mutation information.

Of the 94 missing concept triples in Table 8 for the full set of HITs (444 gold standard triples—350 Turker ‘YES’ responses), 43 were due to incorrect extraction of mutation position information and 37 were due to missing stop codon or synonymous mutations (neither of which are recognized by the version of EMU used for the experiment).

### Cost of curation

**Figure 3.**
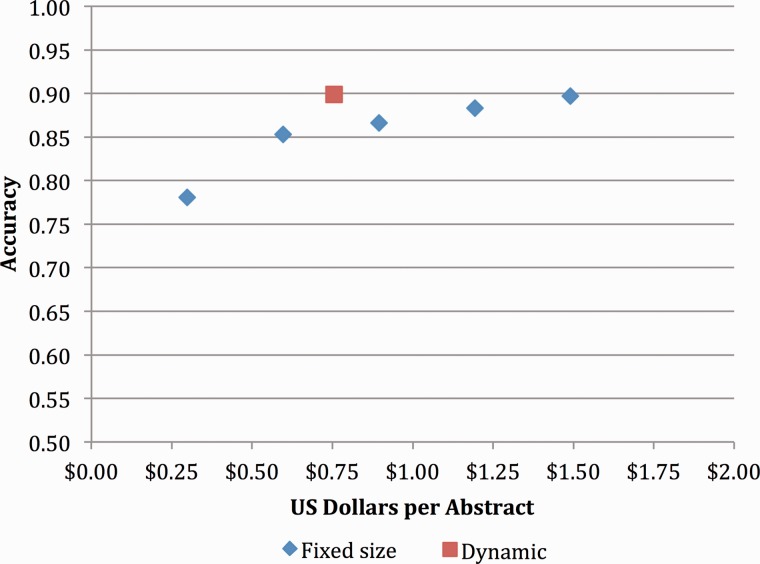
Concept accuracy for static and dynamic Turker pools, removing mutations with nonlocal positions.

The ultimate goal of this research was to develop an accurate, scalable, cost-effective method to capture gene–mutation relations (and ultimately gene–mutation–disease relations) in an open source repository. The initial results of both experiments showed promise in terms of throughput but fell short of the goal of 90% precision at >70% recall. However, when we revisited these results using only mutations extracted with local position information, we saw that the precision improved to 82.3% with a recall of 71.4% (Table 8). Figure 3 shows the cost-accuracy trade-offs for this more limited set of mutations, with the optimal trade-off of 89.9% accuracy at a cost of $0.76 achieved by simulating removal of any Turker whose performance fell below 50% accuracy on the control items.

On the other hand, the system could be biased for high recall with an expert curator inserted to review all judgments before depositing these in a database. While this would add some element of cost, it is much faster to review relations than to tag them de novo. The expert could use a version of the same interface, along with an explicit visualization of the concept level information, specifically the EntrezGene ID, gene symbol and gene name, also representation of the wild type and mutant type amino acid or nucleotide and its position.

These issues point to the need for further experimentation. Open issues include the following:

- Exploration of alternative mutation extraction and gene normalization tools. For example, a recent paper by Jimeno Yepes and Verspoor (33) compared open source tools for automatic extraction of mutations, including (20, 34–38). Their results suggest that a combination of tools could yield higher performance, which could lead to improvements in both precision and recall.
- The adaptation of the current interface for use by expert curators, including explicit representation of concept level output;
- The cost of expert review to validate entries before archiving, with a workflow optimized for recall;
- The cost of capturing more complex relations, e.g. gene–mutation–disease;
- The ability to recruit and retain vetted Turkers to perform these tasks;
- The ability to curate full text articles, as opposed to abstracts. This latter is particularly important, as abstracts contain only a subset of genetic variants; the full text contains a much richer set of variants, and the supplementary material contains even more (39). However, the longer the text segment, the more challenging it becomes to show a useful context to the Turker for relation judgment.

## Conclusions

Overall, we concluded that the hybrid curation approach was extremely promising: it met our goals in terms of cost and turnaround, but fell somewhat short on quality, with accuracy of just <90%, end-to-end recall at an acceptable 71%, but precision still below the target of 90%.

Precision was hurt by discrepancies between instructions and aims of experts preparing the gold standard and the (rather simplified) instructions given to the Turkers. To mitigate this, we could explore several options. The first would be to do Turking in two stages: a first stage would be to judge the correctness of the mutation, and a second stage could judge the correctness of the gene–mutation relation. An alternative option would be to experiment with exposing more explicit mutation information to the Turkers, and asking them to also validate the correctness of the mutation.

If we wished to extend this approach to disease–gene– mutation curation, we would need to adopt a similar approach, to break the problem down into smaller subcomponents that could be separately judged; our early experiments on curation of the three-way disease–gene– mutation relations suggested that this task would be cognitively too complex, with too many ways for a relation to be incorrect.

Overall, we were pleasantly surprised that we were able to recruit Turkers with sufficient ability to handle this complex domain; and even though we were not able to measure Turker surface judgments directly, we estimated that the surface accuracy of the aggregate judgments was around 90%.

Finally, we were encouraged by the rapid turnaround: in the initial experiment, 250 abstracts in 36 h; in the second experiment, 275 abstracts in 11 days over the December holidays. Even more encouraging, if we were to adopt an approach of only paying ‘good’ Turkers, we could achieve 90% accuracy at a cost of only $0.76 per abstract—well below our target of $1 per abstract.

## Competing Interests

The authors declare that they have no competing interests.

## Supplementary Data

Supplementary data are available at *Database* Online.

## Funding

This research has been partially funded by the Intramural Research Program of NIH, National Library of Medicine and by The National Institutes of Health (NIH) grant 1K22CA143148 to M.G.K. (PI) and the American Cancer Society, ACS-IRG grant to M.G.K. (PI). The curation results reported in Supplementary Appendix A (Returking of Random HITS) was performed at University of Maryland, Baltimore County, under the supervision of M.G.K. by Urooj Aqeel, Veer Gariwala, Mitsu Shah, Nikhil Yesupriya and Ye Sol Yun. Funding for open access charge: The MITRE Corporation.

*Conflict of interest*: None declared.

## Supplementary Material

Supplementary Data
