# Supplementary material for: Hybrid curation of gene–mutation relations combining automated extraction and crowdsourcing
Source: Database (Oxford). 2014 Sep 22;2014:bau094. doi: 10.1093/database/bau094 (PMC4170591; doi:10.1093/database/bau094)
Supplement: Supplementary Data [file supp_2014_bau094_index.html]

Hybrid curation of gene–mutation relations combining automated extraction and crowdsourcing — Supplementary Data 

# Hybrid curation of gene–mutation relations combining automated extraction and crowdsourcing

## Supplementary Data

files

**Files in this Data Supplement:**

- Supplementary Data - docx file
- Supplementary Data - png file
- Supplementary Data - docx file
- Supplementary Data - docx file
- Supplementary Data - docx file
- Supplementary Data - docx file
- Supplementary Data - docx file
- Supplementary Data - docx file
- Supplementary Data - docx file
- Supplementary Data - docx file
