# Supplementary material for: Hybrid curation of gene–mutation relations combining automated extraction and crowdsourcing
Source: Database (Oxford). 2014 Sep 22;2014:bau094. doi: 10.1093/database/bau094 (PMC4170591; doi:10.1093/database/bau094)
Supplement: Supplementary Data [file supp_bau094_Appendix_D_v30.docx]

**Appendix D: Instructions and Qualifier Items**

Below we include the instructions given to the Turkers. These were available in a pop up on each item, and also as the introduction to the qualifier exam.

**Instructions**

The task is to read a series of medical abstracts that discuss genes and proteins, as well as mutations. Genes encode information about proteins, and so these are often discussed interchangeably. Mutations occur on genes, but are often described with respect to proteins, because a gene mutation can cause a change in the resulting protein.

You will be asked to judge whether each abstract associates a particular mutation with a specific gene or protein. The abstract may discuss multiple genes/proteins and multiple mutations; it may also mention a particular gene/protein or mutation more than once. You are only being asked about whether the highlighted mutation is associated with the highlighted gene or protein.

You may see multiple sections of text highlighted with the same color; these should be mentions of the same gene/protein or the same mutation, possibly with different wording. If you believe that these are not all the same, please select the option **Inconsistent Annotation**.

A mutation may be highlighted that is not associated with the highlighted gene or protein. If this is the case, then answer **No**.

If you are reasonably certain that the mutation is in fact associated with the gene or protein, then answer **Yes**.

### We also include the five items used for the qualifier. Turkers were required to answer at least four of these items correctly in order to participate in the task. We show the expected answer for each item in bold. We also show the PubMed identifier of each abstract here for reference purposes only—the Turkers were not presented with the PMIDs.

### Commonly studied single-nucleotide polymorphisms and breast cancer: results from the Breast Cancer Association Consortium. (PMID 17018785)

BACKGROUND: The Breast Cancer Association Consortium (BCAC) is an international collaboration that was established to provide large sample sizes for examining genetic associations. We conducted combined analyses on all single-nucleotide polymorphisms (SNPs) whose associations with breast cancer have been investigated by at least three participating groups. METHODS: Data from up to 12 studies were pooled for each SNP (ADH1C I350V, AURKA F31I, BRCA2 N372H, **CASP8** D302H, ERCC2 **D312N**, IGFBP3 -202 c>a, LIG4 D501D, PGR V660L, SOD2 V16A, TGFB1 L10P, TP53 R72P, XRCC1 R399Q, XRCC2 R188H, XRCC3 T241M, XRCC3 5' UTR, and XRCC3 IVS7-14). Genotype frequencies in case and control subjects were compared, and genotype-specific odds ratios for the risk of breast cancer in heterozygotes and homozygotes for the rare allele compared with homozygotes for the common allele were estimated with logistic regression. Statistical tests were two-sided. RESULTS: The total number of subjects for analysis of each SNP ranged from 12,013 to 31,595. For five SNPs--**CASP8** D302H, IGFBP3 -202 c>a, PGR V660L, SOD2 V16A, and TGFB1 L10P--the associations with breast cancer were of borderline statistical significance (P = .016, .060, .047, .056, and .0088 respectively). The remaining 11 SNPs were not associated with breast cancer risk; genotype-specific odds ratios were close to unity. There was some evidence for between-study heterogeneity (P<.05) for four of the 11 SNPs (ADH1C I350V, ERCC2 **D312N**, XRCC1 R399Q, and XRCC3 IVS5-14). CONCLUSION: Pooling data within a large consortium has helped to clarify associations of SNPs with breast cancer. In the future, consortia such as the BCAC will be important in the analysis of rare polymorphisms and gene x gene or gene x environment interactions, for which individual studies have low power to identify associations, and in the validation of associations identified from genome-wide association studies.

Does this abstract indicate that the **mutation** is associated with the **gene/protein**?

🞆 Yes
**🞊** **No**
🞆 Inconsistent annotation

### The 5alpha-reductase type II ****A49T**** and V89L high-activity allelic variants are more common in men with prostate cancer compared with the general population. (PMID 16039774)

OBJECTIVES: To compare men with prostate disease with those from the general population regarding polymorphisms in the **androgen receptor** gene and in the 5alpha-reductase II (SRD5A2) gene. MATERIALS AND METHODS: The SRD5A2 polymorphisms **A49T**, V89L and R227Q, the **androgen receptor** CAG and GGN repeats and sex hormone status was investigated in men with prostate cancer (CaP) (n=89), benign prostate hyperplasia (n=45) and healthy military conscripts (n=223). RESULTS: The SRD5A2 high-activity allele variants **A49T** AT and V89L LL were more frequent in CaP-patients compared to general population, p=0.026 and p=0.05, respectively. CaP progression was, however, independent of SRD5A2 variants. In contrary, men with GGN<23 had a higher risk of dying from the disease than their counterparts with longer repeats. CONCLUSIONS: Men with CaP were more often genetically predisposed to a higher enzymatic activity in the turn over from T to DHT compared to the general population. In our population, **androgen receptor** genotype affected CaP outcome.

Does this abstract indicate that the **mutation** is associated with the **gene/protein**?

🞆 Yes
**🞊** **No**
🞆 Inconsistent annotation

### Suppression of ****PTEN**** function increases breast cancer chemotherapeutic drug resistance while conferring sensitivity to mTOR inhibitors. (PMID 18332865)

Ectopic expression of mutant forms of phosphatase and tensin homologue deleted on chromosome 10 (**PTEN**) lacking lipid (G129E) or lipid and protein (**C124S**) phosphatase activity decreased sensitivity of MCF-7 breast cancer cells, which have wild-type **PTEN**, to doxorubicin and increased sensitivity to the mammalian target of rapamycin (mTOR) inhibitor rapamycin. Cells transfected with a mutant **PTEN** gene lacking both lipid and protein phosphatase activities were more resistant to doxorubicin than cells transfected with the **PTEN** mutant lacking lipid phosphatase activity indicating that the protein phosphatase activity of **PTEN** was also important in controlling the sensitivity to doxorubicin, while no difference was observed between the lipid (G129E) and lipid and protein (**C124S**) phosphatase **PTEN** mutants in terms of sensitivity to rapamycin. A synergistic inhibitory interaction was observed when doxorubicin was combined with rapamycin in the phosphatase-deficient **PTEN**-transfected cells. Interference with the lipid phosphatase activity of **PTEN** was sufficient to activate Akt/mTOR/p70S6K signaling. These studies indicate that disruption of the normal activity of the **PTEN** phosphatase can have dramatic effects on the therapeutic sensitivity of breast cancer cells. Mutations in the key residues which control **PTEN** lipid and protein phosphatase may act as dominant-negative mutants to suppress endogenous **PTEN** and alter the sensitivity of breast cancer patients to chemo- and targeted therapies.

Does this abstract indicate that the **mutation** is associated with the **gene/protein**?

**🞊** **Yes**
🞆 No
🞆 Inconsistent annotation

### Contribution of germline BRCA1 and ****BRCA2**** sequence alterations to breast cancer in Northern India. (PMID 17018160)

BACKGROUND: A large number of distinct mutations in the BRCA1 and **BRCA2** genes have been reported worldwide, but little is known regarding the role of these inherited susceptibility genes in breast cancer risk among Indian women. We investigated the distribution and the nature of BRCA1 and **BRCA2** germline mutations and polymorphisms in a cohort of 204 Indian breast cancer patients and 140 age-matched controls. METHOD: Cases were selected with regard to early onset disease (< or =40 years) and family history of breast and ovarian cancer. Two hundred four breast cancer cases along with 140 age-matched controls were analyzed for mutations. All coding regions and exon-intron boundaries of the BRCA1 and **BRCA2** genes were screened by heteroduplex analysis followed by direct sequencing of detected variants. RESULTS: In total, 18 genetic alterations were identified. Three deleterious frame-shift mutations (185delAG in exon 2; 4184del4 and 3596del4 in exon 11) were identified in BRCA1, along with one missense mutation (K1667R), one 5'UTR alteration (22C>G), three intronic variants (IVS10-12delG, IVS13+2T>C, IVS7+38T>C) and one silent substitution (5154C>T). Similarly three pathogenic protein-truncating mutations (6376insAA in exon 11, 8576insC in exon19, and 9999delA in exon 27) along with one missense mutation (**A2951T**), four intronic alterations (IVS2+90T>A, IVS7+75A>T, IVS8+56C>T, IVS25+58insG) and one silent substitution (1593A>G) were identified in **BRCA2**. Four previously reported polymorphisms (K1183R, S1613G, and M1652I in BRCA1, and 7470A>G in **BRCA2**) were detected in both controls and breast cancer patients. Rare BRCA1/2 sequence alterations were observed in 15 out of 105 (14.2%) early-onset cases without family history and 11.7% (4/34) breast cancer cases with family history. Of these, six were pathogenic protein truncating mutations. In addition, several variants of uncertain clinical significance were identified. Among these are two missense variants, one alteration of a consensus splice donor sequence, and a variant that potentially disrupts translational initiation. CONCLUSION: BRCA1 and **BRCA2** mutations appear to account for a lower proportion of breast cancer patients at increased risk of harboring such mutations in Northern India (6/204, 2.9%) than has been reported in other populations. However, given the limited extent of reported family history among these patients, the observed mutation frequency is not dissimilar from that reported in other cohorts of early onset breast cancer patients. Several of the identified mutations are unique and novel to Indian patients.

Does this abstract indicate that the **mutation** is associated with the **gene/protein**?

**🞊** **Yes**
🞆 No
🞆 Inconsistent annotation

### Down-regulation of BRCA1-BARD1 ubiquitin ligase by CDK2. (PMID 15665273)

BRCA1, a breast and ovarian tumor suppressor, is a phosphoprotein whose cellular expression level is regulated in a cell cycle-dependent manner. BRCA1 interacts with BARD1 to generate significant ubiquitin ligase activity which catalyzes nontraditional Lys-6-linked polyubiquitin chains. However, it is not clear how the activity is regulated and how this affects BRCA1's multiple cellular functions. Here we show that the ubiquitin ligase activity of BRCA1-BARD1 is down-regulated by CDK2. During the cell cycle, BARD1 expression can largely be categorized into three patterns: moderately expressed in a predominantly unphosphorylated form in early G(1) phase, expressed at low levels in both phosphorylated and unphosphorylated forms during late G(1) and S phases, and highly expressed in its phosphorylated form during mitosis coinciding with BRCA1 expression. CDK2-cyclin A1/E1 and CDK1-cyclin B1 phosphorylate BARD1 on its NH(2) terminus in vivo and in vitro. Intriguingly, the BRCA1-BARD1-mediated in vivo ubiquitination of nucleophosmin/B23 (NPM) and autoubiquitination of BRCA1 are dramatically disrupted by coexpression of CDK2-cyclin A1/E1, but not by CDK1-cyclin B1. The inhibition of ubiquitin ligase activity is not due to the direct effect of the kinases on BARD1 because an unphosphorylatable mutant of BARD1, **S148A**/S251A/S288A/T299A, is still inhibited by CDK2-**cyclin E1**. Alternatively, BRCA1 and BARD1 are likely exported to the cytoplasm and their expressions are remarkably reduced by CDK2-**cyclin E1** coexpression. Recognizing the importance of **cyclin E1** overexpression in breast cancer development, these results suggest a CDK2-BRCA1-NPM pathway that coordinately functions in cell growth and tumor progression pathways.

Does this abstract indicate that the **mutation** is associated with the **gene/protein**?

🞆 Yes
**🞊** **No**
🞆 Inconsistent annotation
