# Supplementary figures and images for: Hybrid curation of gene–mutation relations combining automated extraction and crowdsourcing
Source: Database (Oxford). 2014 Sep 22;2014:bau094. doi: 10.1093/database/bau094 (PMC4170591; doi:10.1093/database/bau094)

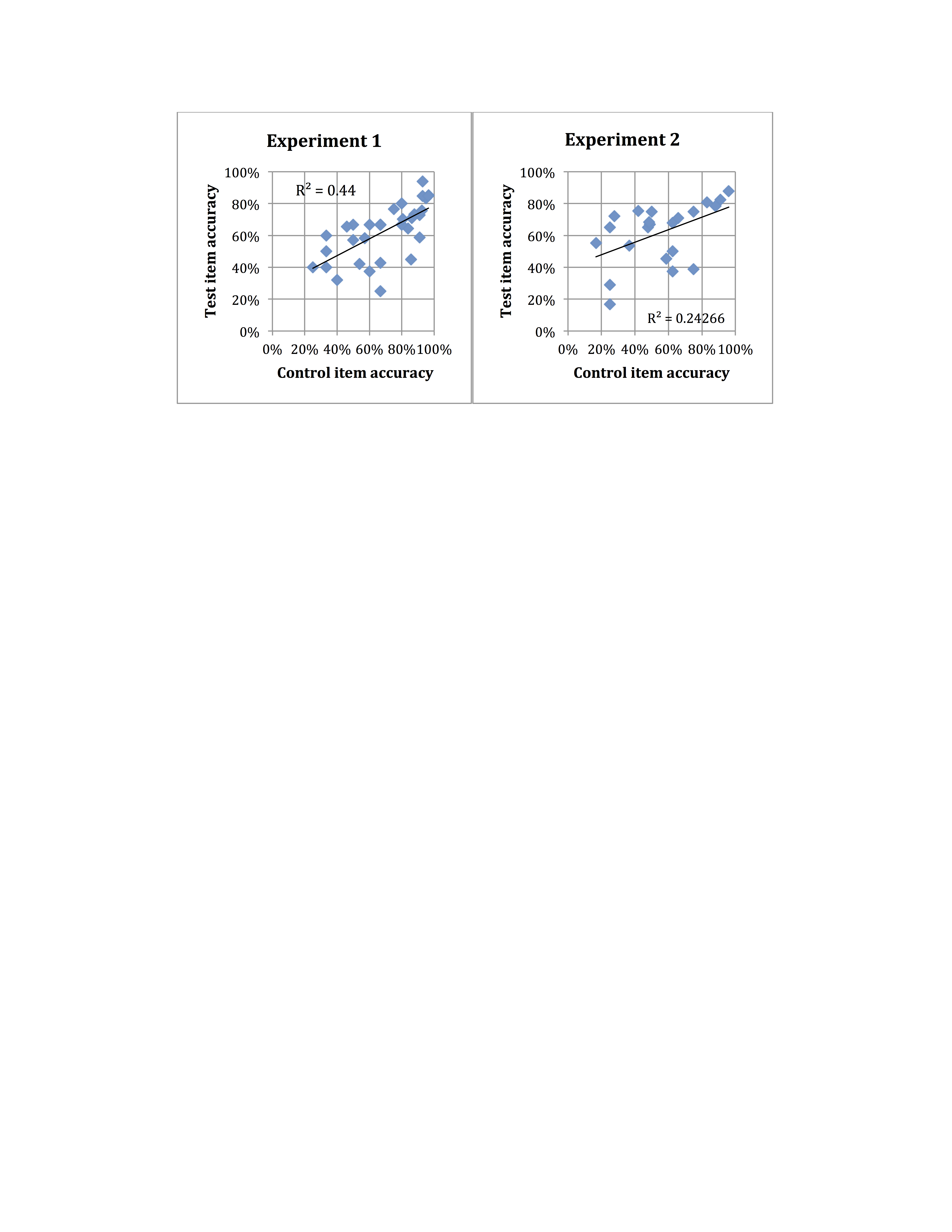

Supplement: Supplementary Data [file supp_bau094_FigureB1.png]
