# Supplementary material for: Hybrid curation of gene–mutation relations combining automated extraction and crowdsourcing
Source: Database (Oxford). 2014 Sep 22;2014:bau094. doi: 10.1093/database/bau094 (PMC4170591; doi:10.1093/database/bau094)
Supplement: Supplementary Data [file supp_bau094_HybridCurationOxDB-v25-AppendixA-formatted.docx]

# Appendix A: Re-Turking Results

We performed two sets of experiments to attempt to provide a ground truth set for surface relation judgments.

## Returking all Turker/Gold Standard Disagreements

In the first experiment, we selected all of the HITS where the aggregate Turker judgment differed from the Gold Standard. Three of the authors (RK, JA, LH) “re-turked” 125 HITs where the aggregate Turker judgment differed from the gold standard (both false positives and false negatives, but done using an earlier version of the gold standard). These three authors performed 119 HITs in common. Response counts appear in Table A1.

[INSERT TABLE A1 ABOUT HERE]

Pairwise raw agreement and Cohen’s Kappa for author re-turked HITs appear in Table A2.

[INSERT TABLE A2 ABOUT HERE]

Although the agreement levels among the three authors (Table A2) was slightly higher than for Turkers (Table 6 in the paper), the agreement levels were not particularly high in either case. However, there were 83 HITs in the set (66%) where all three annotators agreed; and of those, the “re-turking” agreed with the original Turkers (and disagreed with the gold standard) in 60 of those cases.

## Returking a Random Subset of HITS

In the second experiment, six annotators from UMBC provided two-fold judgments of the HITs from a randomly selected subset of 100 abstracts. We performed several analyses on the 383 items that were presented to the UMBC annotators:

1. VirtualTurkers: Analysis using every ItemID, attributing one response to "virtualturker1" and the other to "virtualturker2" (383 items – Table A3)
2. ProlificTurkers: Analysis using the overlap items of the two most prolific Turkers (124 items – Table A4)

[INSERT TABLE A3 ABOUT HERE]

For each of these, we looked at the full task with four possible answers (yes/no/inconsistent/blank) and a binary task (yes/not-yes). For the VirtualTurkers, we calculated just raw agreement, and for the ProlificTurkers we calculated raw agreement and Kappa.

[INSERT TABLE A4 ABOUT HERE]

We see about 60% raw agreement between the virtual UMBC annotators, and 72% agreement for the binary task. This is slightly better than the actual Turkers (Table 7 in the paper). When looking at the actual Prolific Turker results (Table A4), we see that binary task agreement is (not surprisingly) quite a bit higher than full task agreement. For the ProlificTurkers we can also look at patterns of individual Turker tag usage and calculate kappa; Table A4 above shows that full task agreement is 63.7%, with a kappa of 0.40; binary task agreement is 80.6%, with a kappa of 0.61. These results are better than the agreement between actual Turkers and comparable to the best pair of authors.

While the 80% agreement between the two Prolific UMBC Turkers is encouraging, even the best results fall short of the 90% inter-curator agreement achieved by expert curated databases. This may be a reflection of task difficulty, variations in background knowledge, or some other factor; see Snow et al (2008).

# References

Snow R et al. (2008), **Cheap and fast---but is it good?: evaluating non-expert annotations for natural language tasks**, Proceeding EMNLP '08 Proceedings of the Conference on Empirical Methods in Natural Language Processing, pps. 254-263
