# Supplementary material for: Hybrid curation of gene–mutation relations combining automated extraction and crowdsourcing
Source: Database (Oxford). 2014 Sep 22;2014:bau094. doi: 10.1093/database/bau094 (PMC4170591; doi:10.1093/database/bau094)
Supplement: Supplementary Data [file supp_bau094_HybridCurationOxDB-v25-AppendixB-formatted.docx]

# Appendix B: Comparison Between Control and Test

As mentioned above, we inserted control items into the workflow to gauge which Turkers to trust more. In our first experiment, an arbitrary subset of the items was chosen to act as controls. In the current study, we selected items from Experiment 1 that were highly predictive of overall performance, as described in the Methods section above. The graphs in Figure B1 show concept level relation accuracy for individual Turkers on the control items (x-axis) plotted against performance on test items (y-axis). To our surprise, Experiment 1 (Figure B1, left) showed a higher correlation than Experiment 2 (Figure B1, right): an R^2^ of 0.44 for Experiment 1 vs. R^2^ of 0.24 for Experiment 2. The lower correlation for Experiment 2 was unexpected and has led us to re-examine the criteria for selection of control items.

[INSERT FIGURE B1 ABOUT HERE]

Figure B1: Control item performance vs test items
