# Supplementary material for: Hybrid curation of gene–mutation relations combining automated extraction and crowdsourcing
Source: Database (Oxford). 2014 Sep 22;2014:bau094. doi: 10.1093/database/bau094 (PMC4170591; doi:10.1093/database/bau094)
Supplement: Supplementary Data [file supp_bau094_HybridCurationOxDB-v25-AppendixC-formatted.docx]

# Appendix C: Analysis of Turker False Positives

After removing all HITs that had non-local positional information, we analyzed the remaining 68 false positive aggregate Turker judgments. The analysis was performed in terms of the following categories:.

1. **Non-human mutation:** The abstract mentions mutations from a non-human organism, EMU finds these and generates HITs. However, the gold standard flags all non-human mutations and they are excluded from the scoring procedure.
2. **Non-coding mutation:** Only later entries in the gold standard consistently captured non-coding mutations, so non-coding mutations are ignored during scoring. If, however, EMU extracts an apparent coding representation for a non-coding mutation, it may result in a false positive.^[[1]](#footnote-1)^
3. **Missing Gold Standard information:** In a few cases, a mutation may be mentioned at both the amino acid level and the nucleotide level, but the position may only be given in terms of the codon (amino acid). In these cases, the gold standard includes an entry lacking position information for the nucleotide level; thus any nucleotide level mutation that EMU produces will not match the gold standard.
4. **Gene name or gene ID problem**: The gene ID for a correctly highlighted gene doesn’t match the gold standard, with two underlying causes:
   - Incomplete capture of the gene name leading to incorrect gene identifier, e.g., using “insulin-growth factor II” instead of “IGFR2” (where R stands for receptor, making this a different gene);
   - Incorrect selection of the gene identifier in cases of ambiguity.
5. **Erroneous Turker judgment:** The relation was approved despite the Turker being presented with sufficient information to indicate that the proposed relation was incorrect.

[INSERT TABLE C1 ABOUT HERE]

From Table C1, we see that there were categories of errors where there may have been insufficient instructions given to the Turkers (categories 1: non-human or not curated; and 2: non-coding mutations) and/or a mismatch between gold standard and the Turker task (category 3: gene or position not present in the Gold Standard). For example, if Turkers had been instructed to *only* judge mutations occurring in human genes and to ignore any non-coding mutations, this might have eliminated many errors in categories 1 and 2, assuming that Turkers had made correct judgments about whether or not a given abstract contained the appropriate information.

Similarly, a number of the category 4 errors (those relating to gene ID problems) were hidden from the Turkers, who saw a plausible gene name highlighted in the text and may have judged the relation between gene and mutation as correct. Almost 1/3 of the errors involved a hidden error in gene ID.

1. If EMU extracts information positional information that characterizes the mutation as non-coding (e.g., a negative position), then this is filtered out and no HIT is generated for this mutation. [↑](#footnote-ref-1)
