# Supplementary material for: Hybrid curation of gene–mutation relations combining automated extraction and crowdsourcing
Source: Database (Oxford). 2014 Sep 22;2014:bau094. doi: 10.1093/database/bau094 (PMC4170591; doi:10.1093/database/bau094)
Supplement: Supplementary Data [file supp_bau094_Table_A1.docx]

**Table A1: Author responses to re-Turked HITs**

|  | **X** | **Y** | **Z** | **Total** |
| --- | --- | --- | --- | --- |
| **yes** | 81 | 97 | 109 | **287** |
| **no** | 23 | 14 | 8 | **45** |
| **inconsistent** | 15 | 8 | 2 | **25** |
| **Total** | **119** | **119** | **119** |  |
