# Supplementary material for: Hybrid curation of gene–mutation relations combining automated extraction and crowdsourcing
Source: Database (Oxford). 2014 Sep 22;2014:bau094. doi: 10.1093/database/bau094 (PMC4170591; doi:10.1093/database/bau094)
Supplement: Supplementary Data [file supp_bau094_Table_A2.docx]

Table A2: Pairwise Agreement and Kappa for Re-Turked HITs

|  | **X-Y** | **X-Z** | **Y-Z** |
| --- | --- | --- | --- |
| **% Agreement** | 0.765 | 0.739 | 0.849 |
| **Cohen’s Kappa** | 0.432 | 0.279 | 0.381 |
