# Supplementary material for: Hybrid curation of gene–mutation relations combining automated extraction and crowdsourcing
Source: Database (Oxford). 2014 Sep 22;2014:bau094. doi: 10.1093/database/bau094 (PMC4170591; doi:10.1093/database/bau094)
Supplement: Supplementary Data [file supp_bau094_Table_A3.docx]

Table A3: Analysis of "virtual UMBC Turkers"

| **Count** | **Percent** |  |
| --- | --- | --- |
| **4-way task** | | |
| **383** | 100.00% | Items – 4-way task |
| **227** | 59.27% | Agreement |
| **158** | 41.25% | Both-yes |
| **61** | 15.93% | Both-no |
| **8** | 2.09% | Both-inconsistent |
| **0** | 0.00% | Both-blank |
| **Binary task** | | |
| **383** | 100.00% | Items – yes/not-yes task |
| **277** | 72.32% | Binary-task-agreement |
| **158** | 41.25% | Both-yes |
| **119** | 31.07% | Both-not-yes |
