# Supplementary material for: Hybrid curation of gene–mutation relations combining automated extraction and crowdsourcing
Source: Database (Oxford). 2014 Sep 22;2014:bau094. doi: 10.1093/database/bau094 (PMC4170591; doi:10.1093/database/bau094)
Supplement: Supplementary Data [file supp_bau094_Table_A4.docx]

Table A4: Prolific Turker results

| **Count** | **Percent** |  |
| --- | --- | --- |
| **4-way task** | | |
| **124** | 100.00% | Items – 4-way task |
| **79** | 63.71% | Agreement |
| **54** | 43.55% | Both-yes |
| **23** | 18.55% | Both-no |
| **2** | 1.61% | Both-inconsistent |
| **0** | 0.00% | Both-blank |
| **Binary task** | | |
| **124** | 100.00% | Items – yes/not-yes task |
| **100** | 80.65% | Binary-task-agreement |
| **54** | 43.55% | Both-yes |
| **46** | 37.10% | Both-not-yes |
