# Supplementary material for: Hybrid curation of gene–mutation relations combining automated extraction and crowdsourcing
Source: Database (Oxford). 2014 Sep 22;2014:bau094. doi: 10.1093/database/bau094 (PMC4170591; doi:10.1093/database/bau094)
Supplement: Supplementary Data [file supp_bau094_Table_C1.docx]

Table C1: Categorization of false positives (excluding HITs from non-local mutations)

| 1. Non-human mutation | 2. Non-coding mutation | 3. Missing gold standard information | 4. Gene name or ID error | 5. Wrong Turker judgement | Total |
| --- | --- | --- | --- | --- | --- |
| 23 | 11 | 3 | 21 | 10 | 68 |
